# Interpretable machine learning models for hospital readmission prediction: a two-step extracted regression tree approach

**DOI:** 10.1186/s12911-023-02193-5

**Published:** 2023-06-05

**Authors:** Xiaoquan Gao, Sabriya Alam, Pengyi Shi, Franklin Dexter, Nan Kong

**Affiliations:** 1 School of Industrial Engineering, Purdue University, West Lafayette, USA; 2 Department of Electrical Engineering and Computer Sciences, UC Berkeley, Berkeley, USA; 3 Krannert School of Management, Purdue University, West Lafayette, USA; 4 Department of Anesthesia, University of Iowa, Iowa, USA; 5 Weldon School of Biomedical Engineering, Purdue University, West Lafayette, USA

**Keywords:** Hospital readmission, Interpretable machine learning, Risk prediction, Administrative data, Risk factors

## Abstract

**Background:**

Advanced machine learning models have received wide attention in assisting medical decision making due to the greater accuracy they can achieve. However, their limited interpretability imposes barriers for practitioners to adopt them. Recent advancements in interpretable machine learning tools allow us to look inside the black box of advanced prediction methods to extract interpretable models while maintaining similar prediction accuracy, but few studies have investigated the specific hospital readmission prediction problem with this spirit.

**Methods:**

Our goal is to develop a machine-learning (ML) algorithm that can predict 30- and 90- day hospital readmissions as accurately as black box algorithms while providing medically interpretable insights into readmission risk factors. Leveraging a state-of-art interpretable ML model, we use a two-step Extracted Regression Tree approach to achieve this goal. In the first step, we train a black box prediction algorithm. In the second step, we extract a regression tree from the output of the black box algorithm that allows direct interpretation of medically relevant risk factors. We use data from a large teaching hospital in Asia to learn the ML model and verify our two-step approach.

**Results:**

The two-step method can obtain similar prediction performance as the best black box model, such as Neural Networks, measured by three metrics: accuracy, the Area Under the Curve (AUC) and the Area Under the Precision-Recall Curve (AUPRC), while maintaining interpretability. Further, to examine whether the prediction results match the known medical insights (i.e., the model is truly interpretable and produces reasonable results), we show that key readmission risk factors extracted by the two-step approach are consistent with those found in the medical literature.

**Conclusions:**

The proposed two-step approach yields meaningful prediction results that are both accurate and interpretable. This study suggests a viable means to improve the trust of machine learning based models in clinical practice for predicting readmissions through the two-step approach.

**Supplementary Information:**

The online version contains supplementary material available at 10.1186/s12911-023-02193-5.

## Background

### Introduction

Detecting which patients have a greater chance of readmission may allow for better treatment planning during their hospital stays and better follow-up planning after their discharges. In the United States, readmission is a very common problem, with 20% of Medicare beneficiaries readmitted within 30-days after hospital discharge. Readmission costs roughly $17 billion in annual spending [1]. To reduce this significant cost, the Centers for Medicare and Medicaid Services have launched the Hospital Readmissions Reduction Program (HRRP) aiming at reducing the readmission rates [2]. By identifying patients at high risk of readmission, doctors can take targeted interventions to prevent readmission. Further, the prevention of avoidable readmission can significantly improve patient health outcomes and the financial viability of care providers. Traditional tools such as logistic regression and decision tree have the benefit of being easily interpretable, showing which factors play a greater or lesser role in predicting readmission probability. However, the lower accuracy of the traditional models tends to limit their usefulness. Advanced machine learning models such as random forests and neural networks achieve greater accuracy but suffer from limited interpretability [3]. Interpretability is important because such black box methods may have inherent but unknown biases preventing generalizability to different populations. Interpretable models allow practitioners to leverage their clinical knowledge to evaluate and improve the prediction framework.

There is a growing interest in developing interpretable machine learning models, especially in the healthcare outcome prediction contexts. New techniques, such as the one developed in [4], allow one to look inside the black box of more advanced prediction methods to extract interpretable models such as decision trees. These interpretable models offer a similar prediction accuracy as the black box and help identify medically relevant risk factors. The authors of [4] demonstrated success in a diabetic prediction setting. Most existing studies on interpretable machine-learning tools in healthcare focus on supporting medical decision-making, such as [5–7]. Few studies specifically looked into the areas of hospital readmission prediction. It is unclear whether existing interpretable techniques can be successfully applied to this readmission setting, which motivates us to answer the following research question in this paper: in predicting the 30-day and 90-day hospital readmission, can interpretable models maintain good prediction accuracy while increasing interpretability compared to the black box machine learning models?

In this paper, we develop a readmission prediction model that combines the high accuracy of a complex model with the interpretability of simpler models. This methodology applies a two-step process proposed in [4], which trains black box machine learning models for high accuracy and then extracts interpretable regression trees from the final results. The main innovation our paper improves over [4] is to use the continuous scores predicted from the first step regarding the readmission probability to train a regression tree in the second step (in contrast to using binary outcomes from the first step (e.g., readmitted/not readmitted) to train a decision tree as in the original paper [4]). Using data from a large teaching hospital in Asia, we show that this approach greatly improves the accuracy of the extracted tree in the readmission setting, compared with the original method in [4]. We further compare the prediction accuracy from the extracted tree model with that from the black box models, such as neural networks, to quantify the accuracy differential between the interpretable and black box models. Finally, we assess whether decisive factors from the extracted trees are consistent with those from the previous medical literature, confirming that the two-step model does indeed provide interpretable results.

Beyond the technical contribution, our paper contributes to the healthcare operations management literature by demonstrating the potential of recent methodological developments from interpretable machine learning to impact healthcare decision-making, providing evidence from the hospital readmission risk prediction domain. Not only is readmission prediction and prevention of critical importance to hospital operations and finance, but also this use case provides proof of value for interpretable machine learning that can be more broadly applied to the development of predictive models in other healthcare settings to support decision making in healthcare operations.

### Relevant work

Early studies (prior to 2011) on readmission risk analysis used descriptive, particularly discriminatory, analyses to determine the influence on a certain disease or disease classes of a few pre-selected risk factors (or handcrafted features based on experience), including comorbidity [8, 9], age [10–13], sex [14], income [15], and level of education [16], health utilization [11, 12, 16, 17], type of insurance [17, 18]; and treatment and clinical factors [10, 12, 19]. For more details, we refer to a systematic review prepared by the Veterans Health Administration [20], which summarized 26 unique studies presented in English. This review paper notes that these studies often rendered c-statistics ranging from 0.55 to 0.8, with lower values from studies based on administrative data. Our paper also uses administrative data which lacks detailed clinical features. Typical AUCs (one type of c-statistic) for models trained on administrative data are in the range of 0.6–0.7 [20].

To ensure interpretability in hospital readmission prediction, indexing/scoring models have been developed based on predictive variables that can be easily extracted from electronic health records and medical claims data. For example, the LACE index uses four variables -- Length of stay (L), Acuity of the admission (A), Comorbidity of the patient (C), and Emergency department use in the previous six months before admission (E) -- to predict the risk of nonelective 30-day readmission after hospital discharge among both medical and surgical patients [21]. Similarly, the HOSPITAL score uses seven clinical predictors, available in electronic health records, to identify patients at high risk of potentially avoidable hospital readmission within 30 days [22]. Despite some success in identifying key influential risk factors on hospital readmission, these regression models achieve only modest prediction accuracy [21].

On the other hand, there is also a substantial body of literature using machine learning to predict hospital readmission. For example, decision trees, neural networks, logistic regression, and Naïve Bayes classifiers were compared for predicting rehospitalization [23, 24]. With each patient represented by a vector of about 4000 dimensions, a generalized additive model was applied to predict the hospital readmission risk of a general cohort of patients [25]. A comprehensive comparative study with several machine learning methods (support vector machine, decision trees, random forests, and generalized boosting machine) was conducted for predicting all-cause hospital readmission based on administrative data [26]. More recently, deep learning has attracted the attention of the research community on hospital readmission prediction. Several studies have explored its potential [27–29]. Despite success in improving prediction performance over classical regression modeling techniques, all deep-learning models were found to be less suitable for deployment in real-world applications due to the lack of interpretability [30].

Learning interpretable models is challenging because interpretability and accuracy are generally two competing objectives, i.e., one favoring simplicity and generalization while the other favoring nuance and exception. A long-standing question in the field is how to create predictive models that are sufficiently accurate and interpretable for decision making in various applications, e.g., recidivism prediction for sentencing [31], review rating prediction for personalized recommendations [32], and academic performance prediction for university students under warning and probation [33]. Many such studies on interpretable prediction were inspired by medical decision-making. Frank et al. [34] presented a novel method for creating data-driven scoring systems called a Super sparse Linear Integer Model (SLIM). Their experiments demonstrated that an optimized SLIM can create a highly tailored scoring system for sleep apnea screening, breast cancer detection, and survival prediction after breast cancer surgery. Zeng et al. [35] provided a Bayesian framework for learning classification models consisting of an ordered list of if-then rules, which were called falling rule lists. Patients were then stratified into decreasing risk sets with the prediction model built on falling rule lists. Seo et al. [36] introduced generative Bayesian Rule Lists (BRL), which employed a novel prior structure to encourage sparsity. The BRL preserved similar interpretability with scoring systems in practical use but is more accurate. The aforementioned work is focused on generating risk classifications based on sets of binary decision rules, i.e., rules that can be phrased as yes/no questions, providing simple heuristics that are designed to support decision making in settings where the decision-maker has limited time and background knowledge. These decision rules are directly learned from “real” datasets available for training, which are often limited in the sample size. Our work focuses on a setting where the datasets are augmented by “synthetic” data generated from accurate prediction models. In other words, we create a model that mimics the performance of more accurate black box methods and provides medical reasoning behind the predictions. The goal is to increase adoption by instilling confidence in the prediction as well as enabling medical professionals to check the validity and generalizability of these prediction models based on their own clinical knowledge.

## Methods

### Dataset

We used an archived dataset obtained from a major teaching hospital in Southeast Asia, spanning from May 2010 to March 2011. The Purdue IRB (Institutional Review Board) decided that this study of de-identified, archived data does not meet the regulatory definition of human subjects research. The dataset contains a record of 58,036 patients, detailing their medical information including hospital length of stay; insurance class (private versus public insurance); admission source (admitted for elective surgery or from the emergency department); discharge location; medical specialty, and admitting service including month, year, and day of the week of patient admission; patient’s Charlson score; patient’s van Walraven score (see [28]); whether the patient was admitted to the intensive care unit; whether surgical operations were performed; and the number of transfers between intensive care units and general wards. The medical specialties were classified by the hospital; see [29] for a detailed explanation.

### Data pre-processing

The most common specialties represented in this data set were medicine 24.8%, surgery 19.7%, orthopedics 9.5%, and cardiology 9.4%. Among the remaining 36.6% (see Table S1 in the Appendix for these specialties), we excluded pediatrics and obstetrics/gynecology specialties from our prediction analysis. Furthermore, in the prediction analyses, we considered two model settings: one including patients from all the specialties, referred to as the all-specialty model, and one only including patients from the four specialties with the most patients (medicine, surgery, orthopedics, cardiology), referred to as the main-specialty model. Additionally, we tried to standardize or normalize the features. The prediction performances did not have a statistical difference between models with and without feature scaling. As a result, in the subsequent experiments, we used the original un-scaled features.

### Descriptive statistics and supporting data

Table S1 in the Appendix shows the descriptive statistics of the main features of the dataset after the pre-processing. One feature suggested by the literature to improve prediction accuracy was the number of previous hospitalizations for the patient in the last six months prior to the current hospital visit. We added this supporting feature (number of prior visits) via linking to another dataset from the same hospital, which contained patient encounter information from November 2008 to August 2011. Mathematically, denote the admission and discharge date of patient $i$’s $k$th hospital visit as ${{t}^{0}}_{i,k}$ and ${t}_{i,k} , i=1,...,N, k=1,..., {K}_{i}$ respectively, where N is the total number of patients included in the prediction analysis, and ${K}_{i}$ is the maximum number of visits patient $i$ has. Define $h\left(.\right)$ to be the indicator function, which takes value 1 if the indicator is true and 0 otherwise. Then for the record at ${{t}^{0}}_{i,k}$, the number of prior visits within the last six month is calculated as ${\sum }_{j=1}^{k}h({{t}^{0}}_{i,k}-{t}_{i,j} \le 180)$ .

### Predictive targets

For each patient, we were able to use his/her patient ID and visit ID (both deidentified) to calculate the days between hospital admissions. From this variable, we calculated (i) a binary readmission indicator to label whether the patient had a subsequent hospital admission after the current visit, and (ii) the time between two consecutive hospital visits. Following medical convention, we considered both 30-day and 90-day readmission rates as our predictive targets. Mathematically, for the record at ${t}_{i,k}$ (using the same notations as defined above), the two main prediction outcomes can be represented by

- ${{y}_{i,k}}^{30}=h({{t}^{0}}_{i,k+1}-{t}_{i,k}\le 30)$ for the 30-day readmission indicator, and
- ${{y}_{i,k}}^{90}=h({{t}^{0}}_{i,k+1}-{t}_{i,k}\le 90)$for the 90-day readmission indicator.

Rather than make predictions on the general readmission status of a patient, separate models can be created to predict whether a patient is at risk for readmission within these specified windows of time.

**Fig. 1:**
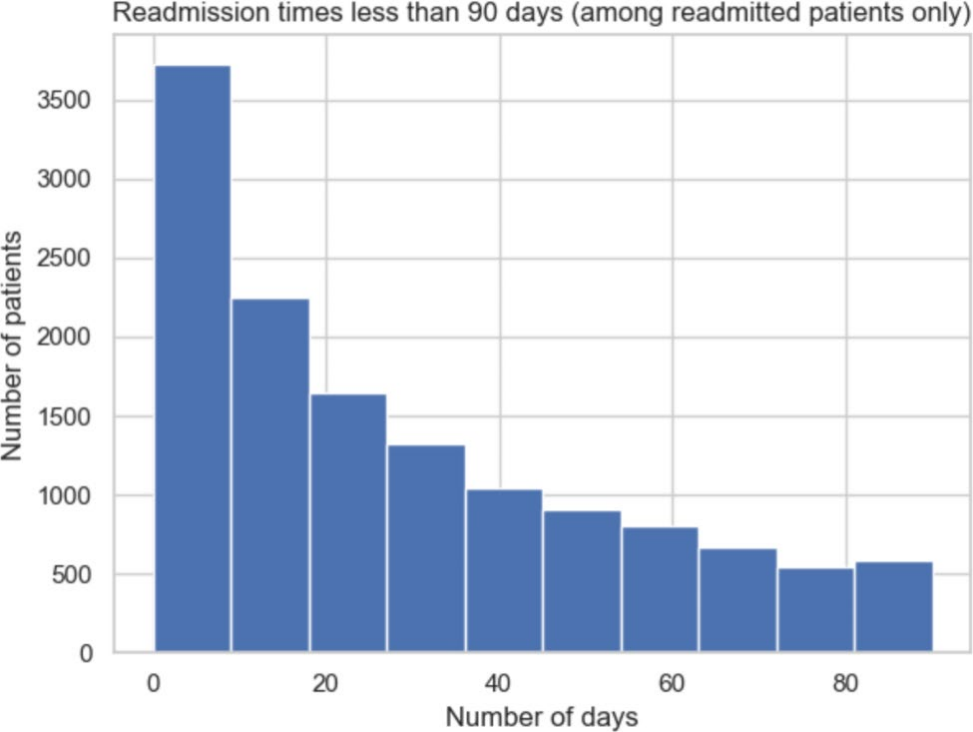
Distribution of the number of patients who are readmitted within 90 days of discharge

Our data shows that 14.3% of patients were readmitted to the hospital within 30 days and 24.4% of patients were readmitted to the hospital within 90 days. Thus, most readmission cases occurred before 30 days have elapsed since the previous visit (Fig. 1). We limited our consideration to the 90-day time window when accounting for readmission because some readmissions beyond 90 days may have causes unrelated to the initial case of hospitalization. In the prediction analysis, rather than making predictions on 30-day and 90-day readmission status together, we created separate models to predict whether a patient is at risk for readmission within each specified window of time.

### Black box model training

For machine learning models, we tested the following: logistic regression (LR), decision tree (DT), support vector machine (SVM), extremely randomized trees (ET), light gradient boosting machine (LGBM), extreme gradient boosting (XGB), random forest (RF), and neural network (NN). We chose logistic regression as the baseline model when comparing the prediction performance. Among these machine learning models we tested, random forests and neural networks have several hyperparameters to tune (e.g., tree depth in the random forest and number of hidden layers in the neural network). For the model tuning, cross validation with stratified k folds was implemented. During each iteration of the k-fold cross-validation process, we split the data into k subsets, trained the model on k-1 subsets, and tested it on the remaining subset. This helped us ensure that the model was not overfit to a specific training and testing set. By repeating this procedure k times and averaging the performance scores obtained over the iterations, we were able to identify a set of hyperparameters that yielded consistently accurate predictions across the k folds. In our implementation of the stratified k-fold, we added an additional step of up- or down-sampling to account for imbalanced class distributions. This approach is especially relevant in classification tasks given that our samples (readmission versus non-readmission) were significantly imbalanced; e.g., 14% 30-day readmitted vs. 86% non-readmitted patients.

### Interpretable machine learning model: two-step extract tree

In order to obtain an interpretable basis for the black box models, we leveraged the cutting-edge ExtractTree algorithm developed in [4]. In this method, any black box machine learning model can be processed with the ExtractTree algorithm to extract representative decision trees. These extracted trees outline which features of the dataset provided insight for the model in making predictions. The process has two steps.

The first step is to learn a good black box prediction model (e.g., train a neural network model). Let ${X}_{train}\in {R}^{n\times d}$ be the feature matrix of the training data, where the $d$ features of the ${i}^{th}$ sample are represented by the ${i}^{th}$ row, ${X}_{i}$. Let ${Y}_{train}\in {R}^{n}$ be the vector of outcomes for the $n$ samples; in our setting, the outcomes are the 30- or 90-day readmission binary indicators as proposed by the original paper [4] (which we will replace by continuous scores in this paper as explained later). Denote the feasible set of the feature space as ${X}_{train}\subseteq X$) and the set for the outcome space as ${Y}_{train}\subseteq Y$. Then we can represent the trained black box model as a function that maps from the feature space to the outcome space, i.e., $f: X\to Y$. Once we train a good model $f$, for any given feasible point $x\in X$ , this mapping provides a prediction $\widehat{y} = f\left(x\right)$.

The second step is to extract interpretable trees using synthetic data labeled by the black box model, i.e., to approximate $f$ using a tree $T$. The tree is a function $T: X\to Y$ as well. For a decision tree (which is used in the original paper [4]), $Y$ takes binary values, i.e., $Y=\{\text{0,1}{\}}^{n}$. For a regression tree that we propose to use this paper, $Y$ takes continuous values, i.e., $Y=[0, 1{]}^{n}$. Specifically, we modify the procedure in the second step for this paper.

- First, we fit the original feature data ${X}_{train}$ with a Gaussian mixture model (GMM) to estimate the joint feature distribution $P$ over inputs from $X.$ This GMM normalizes the features into a distribution from which more feature data could be sampled. We follow [4] and use an expectation-maximization (EM) algorithm to fit the model $P$:

$${p}_{P}\left(x\right)={\sum }_{i=1}^{K}{\varphi }_{i }N({\mu }_{i}, {\varSigma }_{i})$$

where ${p}_{P}$ is the probability density function associated with the distribution $P$, the weights $\varphi \in [0, 1{]}^{k}$ satisfy ${\sum }_{i=1}^{K}{\varphi }_{i}=1$, and the ${i}^{th}$ Gaussian distribution in the mixture has mean ${\mu }_{i}\in {R}^{d}$ and a diagonal covariance matrix ${\varSigma }_{i}\in {R}^{{d}^{2}}$.

- Then, we sample data from this fitted distribution $P$ and run sampled data through the neural network to generate output predictions. That is, we create synthetic data that is generated from $\tilde x \sim {\rm{P}}$ and use the black box model trained in the first step to generate the outcome score, $\tilde{y} = f\left(\tilde{x}\right)\in \left[\text{0,1}\right]$, corresponding to the readmission probability. With the trained model, we can generate as many $(\tilde{x}, \tilde{y})$ pairs as needed. Having access to a large amount of sampled data improved the training performance of the decision tree and regression tree, which otherwise achieved poor performance on the original data (see Table 1).

In [4], the authors extracted decision trees. The major improvement we made in this paper was to apply the same procedure but extract regression trees instead of decision trees. To explain our rationale, though the predicted labels for the data from the neural network are indicated to be 0 (“not readmitted”) or 1 (“readmitted”), the model in fact outputs a continuous score between 0 and 1 (representing a probability of readmission). Thus, to generate the binary prediction, a properly chosen threshold is used to convert the continuous score, where the default threshold is 0.5. That is, if the predicted (continuous) score is below the threshold of 0.5, the predicted label is set to be 0 (non-readmission); otherwise, 1 (readmission). By employing a regression tree, we can directly train on the continuous values predicted by the black box model, rather than the binary values of 0 or 1. This allows us to retain more information from the black box model and improve the accuracy of our interpretable model.

### Performance metrics and model evaluation

Data analyses were performed with scripts written in Python. Machine learning backends used were Keras and Tensorflow, and Python libraries used for modeling and visualization included Sci-Kit Learn, Pandas, and Matplotlib. We adopted the following metrics to compare prediction models: Area Under the Curve (AUC) of the receiver operating characteristic (ROC), area under the precision-recall curve (AUPRC), accuracy (ACC), precision, recall, F-value, and Matthews correlation coefficient (MCC). To assess the model’s ability to generalize to new data, we present the results of out-of-sample testing. This involves evaluating the model on data that it has not seen during training, in order to determine its performance on new, unseen data. We used the performances of the standard logistic regression (LR) as benchmarks to compare the machine learning tools, as it is interpretable and has decent prediction performance.

The experimental results indicate that the relative rankings of the tested methods are mostly consistent in different performance metrics; see Table S2 in the Appendix for the detailed results. In the main paper (“Results” section), we present AUC and AUPRC as well as accuracy, because these three metrics are the most used metrics for classification with unbalanced data. Specifically, accuracy is calculated as the proportion of examples in the test dataset that were predicted correctly, divided by all predictions that were made on the test dataset. The ROC curve plots the false positive rate against the true positive rate for a binary classifier. A more accurate classifier has an ROC curve closer to the top left corner, and AUC closer to 1. An AUC (Area Under the Curve) of 0.5 represents a classifier “guessing” randomly between binary outputs, so an effective classifier must have an AUC higher than the threshold of 0.5. The precision-recall curve is a plot of the precision (the fraction of true positives among all predicted positives) on the y-axis and the recall (the fraction of true positives among all actual positives) on the x-axis. A classifier with a high AUPRC can correctly identify many true positive instances, which is particularly useful in medical diagnosis. Different from AUC, the baseline (actual) AUPRC is equal to the fraction of positive values. For our problem, it was 0.143 (0.244) for 30-day (90-day) prediction in model with all specialties; 0.135 (0.224) for 30-day (90-day) prediction in model with main specialties. In addition to these metrics, we also report the confusion matrices in Table S3 in the Appendix.

To evaluate the out-of-sample testing performance, we leveraged the bootstrapping method to produce the standard errors on the performance scores [30]. That is, for a specified structure of the prediction model and a specified set of its hyperparameters, we ran 50 replications on our dataset where, in each replication, we randomly split the dataset into training and testing according to a 70–30% ratio. We then fit the prediction model, using the specified structure and hyperparameters, on the training data and evaluated the performance on the testing data using the fitted model. This method provided 50 performance scores from one prediction model, allowing us to report the mean performance score and the corresponding sample standard deviation. Because the one-sided upper 95% confidence limit on the normally distributed population standard deviation equals 1.2017 times the sample standard deviation with n = 50, we treated 1.2017 times the standard deviations of the replicated means as deliberately conservative standard errors. The unadjusted standard deviation (SD) of the logistic regression (baseline) model’s accuracy, AUC and AUPRC were measured to be 0.0093, 0.0088, and 0.0049, respectively. This yielded upper confidence limits for the SD of 0.0112, 0.0106, and 0.0058, respectively, when we compared other models against the baseline model. Since multiple comparisons were made, any differences greater than twice the adjusted-SDs were considered statistically significant at a 95% level of confidence (i.e., 0.0224 for accuracy, 0.0212 for AUC, and 0.0116 for AUPRC) and any differences greater than three times the adjusted-SDs were considered statistically significant at a 99% level of confidence (i.e., 0.0336 for accuracy, 0.0318 for AUC, and 0.0174 for AUPRC).

## Results

### Black box model performance

Table 1 below report the prediction performances for the baseline and black box machine learning models on the 30-day and 90-day readmission predictions for the all-specialty and main-specialty models. The baseline LR model yielded an out-of-sample accuracy of 0.706 (0.699), AUC of 0.661 (0.664), and AUPRC of 0.217 (0.339) for 30-day (90-day) readmission predictions. When the subset of patients from the four main specialties was analyzed, the LR model yielded an out-of-sample accuracy of 0.636 (0.664), AUC of 0.621 (0.642), and AUPRC of 0.180 (0.325) for 30-day (90-day) readmission predictions.

The results in Table 1 show that the neural network (NN) model outperformed the logistic regression (LR) model in terms of AUC in all four tested models with a 99% level of confidence. Additionally, the NN model produced a similar or better performance than the LR model for accuracy and AUPRC. Other models, such as Light Gradient Boosting Machine (LGBM), Support Vector Machine (SVM) and Random Forest (RF), also demonstrated significant improvements in accuracy, AUC or AUPRC in some of the tested models as compared to the LR model. While significant performance improvement in the performance scores suggested superior performance in predicting hospital readmissions to LR, this came at the expense of interpretability. Given that NN has the best or close-to-best performance consistently among all the tested models, we chose it as the black box model to extract the interpretable models. The details of the hyperparameters used in the NN model are reported in Sect. 3 (Hyperparameter Settings) in the Appendix.

**Table 1:** Average performance scores of readmission classification (or prediction) models with data rebalancing

Model		30-day			90-day		
		ACC	AUC	AUPRC	ACC	AUC	AUPRC
All Specialties	DT	0.608	0.606	0.181	0.604	0.603	0.288
	LR	0.706	0.660	0.216	0.699	0.664	0.339
	LGBM	0.702	0.683*	0.230*	0.702	0.685	0.355
	ET	0.662	0.660	0.211	0.664	0.668	0.335
	SVM	0.696	0.661	0.215	0.661	0.651	0.332
	RF	0.698	0.688*	0.232*	0.697	0.691*	0.358*
	XGB	0.697	0.674	0.224	0.695	0.678	0.348
	NN	0.708	0.711**	0.213	0.662	0.713**	0.329
Main Specialties	DT	0.574	0.573	0.158	0.571	0.566	0.274
	LR	0.636	0.621	0.180	0.652	0.642	0.325
	LGBM	0.669**	0.655**	0.200*	0.664	0.655	0.336*
	ET	0.625	0.638	0.188	0.643	0.646	0.327
	SVM	0.650	0.638	0.189	0.689**	0.670*	0.341
	RF	0.640	0.650*	0.195*	0.651	0.657	0.334
	XGB	0.626	0.631	0.184	0.641	0.637	0.320
	NN	0.619	0.654**	0.178	0.649	0.685**	0.320

Performances that are determined to be statistically better than the baseline (logistic regression, LR) were marked with a single-asterisk(*) to indicate a significant difference at a 95% level of confidence, and marked with a double-asterisk (**) to indicate a significant difference at a 99% level of confidence. Here, “DT” = decision tree, “LGBM = “light gradient boosting machine”, “ET"= extremely randomized trees, “SVM” = support vector machine, “RF” = random forest, “XGB” = extreme gradient boosting, and “NN” = neural network

### Performance of extracted trees

**Fig. 2:**
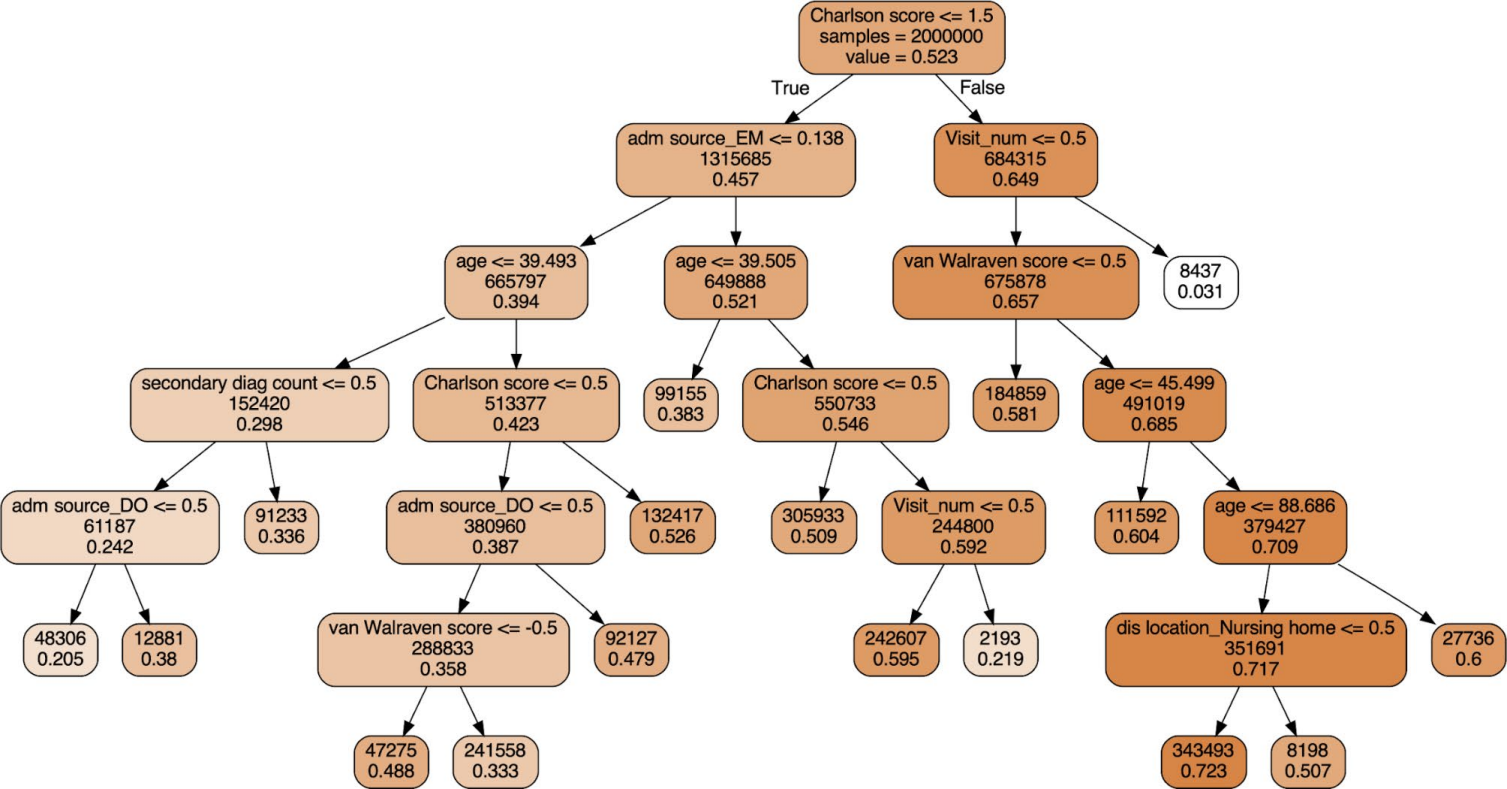
Regression tree extracted from sampled data (Four main specialties, 90-day readmission prediction)

Based on the ExtractTree algorithm outlined in Sect. 3, we extracted decision trees (as in the original paper [4]) and regression trees (our improvement) from the tuned neural network. Table 2 summarizes the performance of (i) Benchmark: the logistic regression model, (ii) Decision tree extracted through the original learning strategy [4], and (iii) Regression tree extracted using our improved strategy [4]. One of the extracted regression trees is shown in Fig. 2. Each node in the regression tree represents a binary split. The intensity of the color at a node corresponds to the proportion of examples classified as “readmitted”. For continuous variables, the split is binary with cutoff values: Charlson score, van Walraven score, number of prior visits (referred to as visit_num in the figure), age, and number of second diagnosis counts. For binary variables, 0.5 is the cutoff value to differentiate between choices: whether the admission source is from the emergency department (EM), and whether the discharge location is to a nursing home.

The decision tree extracted from the neural network model performed no better than the logistic regression model for all four models. In contrast, the regression tree preserved the performance superiority of the neural networks and performed significantly better especially in terms of AUC. Table 2 presents the results of using an adapted version of the ExtractTree algorithm [4], which extracts regression trees instead of decision trees. The results show that this algorithm led to a significant performance improvement in AUC compared to the baseline logistic regression model at a 99% level of confidence.

As discussed earlier, the reason that the extracted regression tree archived a much better performance than the extracted decision tree is that more information is preserved during the synthetic data generation process. That is, when extracting the decision tree, the predictive label we generated is a binary variable, which requires a properly chosen threshold to convert the continuous score from the trained neural network to the binary variable. This adds one layer of information loss. In contrast, by employing a regression tree, we can skip this thresholding step and directly use the continuous score from the trained neural network. Hence, we were able to retain more information from the black box model and improve the accuracy of our interpretable model. It is worth noting that decision tree directly trained on the data (without using the two-step procedure) yielded a worse performance than the LR (see Table 1) and a much worse performance than the extracted RT. For example, the AUC score for 30-day readmission prediction with all specialties is only 0.606 using the directly trained decision tree. Thus, the two-step extraction procedure is the key to improving the predictive performance.

**Table 2:** Average performances of decision trees (DT) and regression trees (RT) extracted from neural networks

		30-day			90-day		
		ACC	AUC	AUPRC	ACC	AUC	AUPRC
All Specialties	LR	0.706	0.660	0.216	0.699	0.664	0.339
	Extracted DT	0.730	0.669	0.226	0.667	0.653	0.325
	Extracted RT	0.729	0.699**	0.227*	0.682	0.697**	0.328
Main Specialties	LR	0.636	0.621	0.180	0.652	0.642	0.325
	Extracted DT	0.614	0.641	0.188	0.639	0.646	0.326
	Extracted RT	0.623	0.648*	0.188	0.643	0.677**	0.322

Performances that are determined to be statistically better than the baseline (logistic regression, LR) were marked with a single-asterisk(*) to indicate a significant difference at a 95% level of confidence and marked with a double-asterisk (**) to indicate a significant difference at a 99% level of confidence

## Discussion

From these results, we can verify our main hypothesis: the extracted regression tree can achieve a similar performance score, compared to the tuned neural network models, while maintaining the important feature of interpretability. A standard decision tree model did not perform better than even the classic logistic regression model. The extracted regression trees improved over the existing models for predicting readmission while providing interpretability.

**Fig. 3:**
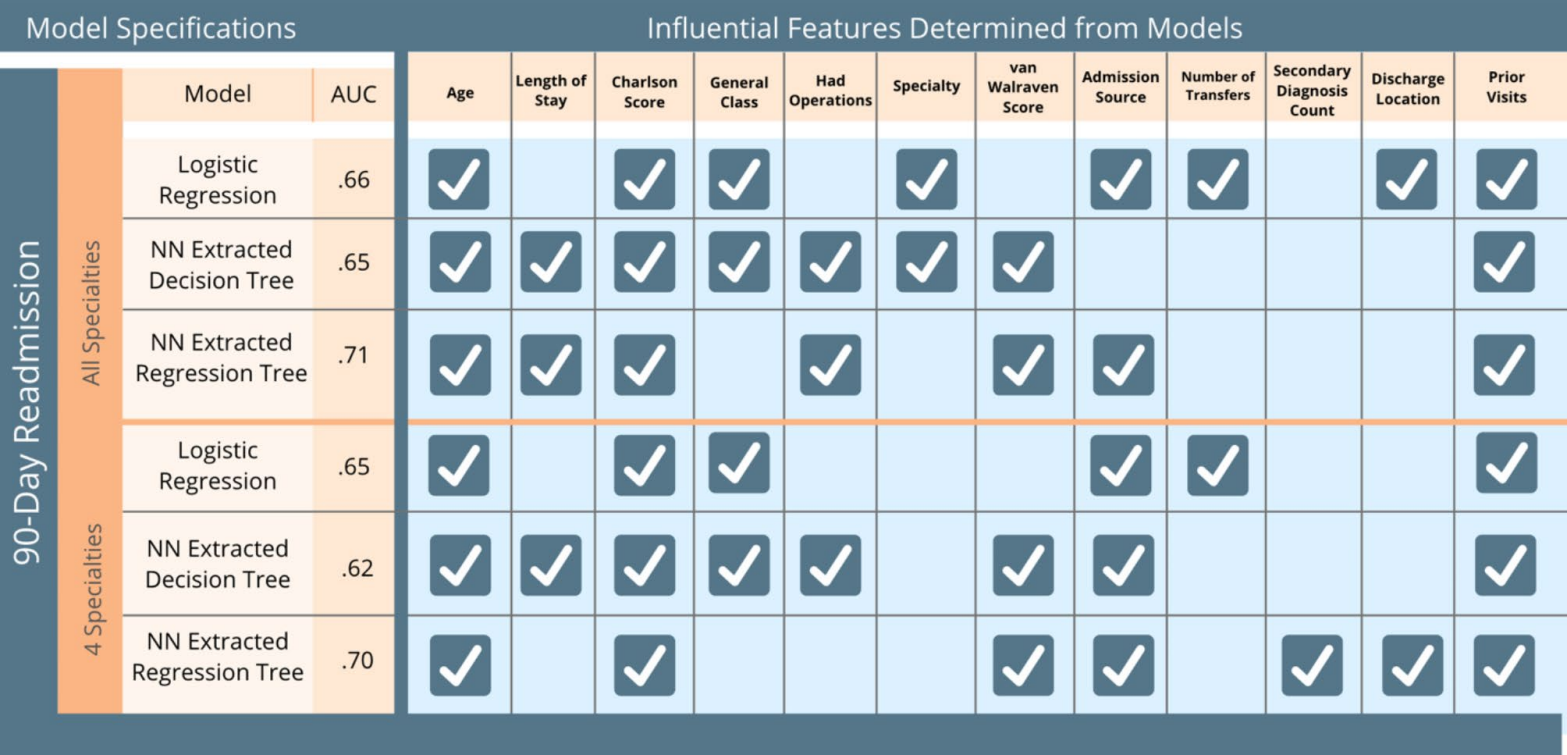
Comparison of Features in Extracted Decision and Regression Trees from black box models on 90-day readmission rate. “General Class” in the table means patient with public insurance. The checkmark means the feature is identified as an influential feature from the corresponding machine learning models

Besides the regression tree extracted from a neural network model for 90-day readmission with all specialties (Fig. 2), we also extracted decision trees and regression trees for the 30- and 90-day readmission predictions with all specialties and main specialties (see the Appendix). Commonalities yielded from a comparison of the generated trees (Fig. 3) implied that the identified features are highly predictive of patients’ readmission status. If decision trees and regression trees extracted from neural network models have overlaps in the node features (indicating the features are influential), then those suggested features may be assessed by subject area experts to determine whether the black box model predictions match clinical knowledge and experience and provide additional features for clinical consideration.

From Fig. 3, we observe that variables reliably important and used in all the models are patient age, Charlson score, admission source, the number of prior visits, and length of stay. Variables in the data but included in none of the models shown were the date of patient admission and the number of transfers between intensive care units and general wards. The variables identified to be important factors were consistent with prior clinical studies, suggesting the extracted trees provide interpretability that can be used by medical doctors.

Historically, readmission prediction models have fallen into two categories: interpretable models with moderate predictive power and non-interpretable machine learning models with strong predictive power. Our contribution is to design a readmission prediction model that achieves similar predictive power to non-interpretable models while being able to generate interpretable features, i.e., identify risk factors that contribute to readmission risk. Adoption of readmission risk prediction tools in practice has been slow, possibly because of the poor prediction of interpretable models that can be validated and integrated with clinical knowledge and the fact that black box models producing better prediction results are difficult to work with as they normally do not naturally offer clinical justification for the predictions. In the end, interpretable models not only help garner trust from users when key risk factors match clinical knowledge but, equally importantly, they provide an opportunity for a closer connection between machine prediction and human (clinical in this case) knowledge and experience.

We calibrated and validated a number of predictive models using data from a large hospital in Southeast Asia. The interpretable two-step method we applied to extract a regression tree from a neural network model had similar performance to the best neural network model, while also being able to identify readmission risk factors. As a validation of the extraction method, the features identified by the extracted regression tree were similar to features found to be predictive of readmissions in the literature. Some key factors predicting readmissions from our model were common, including age, Charlson and van Walraven scores, admission source, and the number of prior visits. For the all-specialty model, Length of Stay and whether the patient had an operation (surgical procedure) were also significant. These factors make sense when considering all specialties, since knowing the length of stay and whether the patient was a surgery patient can serve as proxies for a more detailed classification of the patient type, whereas the main-specialty model had already segmented the population into more specific patient types. When only including the top four specialties, secondary diagnosis and discharge location were also significant, which could help the model further differentiate among patients of a similar type. A key insight is that by using regression trees instead of decision trees we were able to significantly improve the accuracy without losing interpretability because regression trees used more of the information output from the neural network and were, therefore, better able to match risk factors to readmission outcomes.

To conclude the discussion, we acknowledge the following limitations of this work. First, our method was tested on a dataset from a single hospital. While detailed, patient-specific datasets in healthcare are difficult to obtain, further testing in a wider variety of hospitals would better demonstrate the generalizability of these results and represents an avenue for future work. Second, our dataset contains primarily administrative data. Additional clinical data has the potential to improve the black box (e.g., neural network) model. Clinical data is even more difficult to obtain than administrative data, but testing the method proposed in this paper on a richer dataset would be a fruitful avenue for future research.

## Conclusions

The study of readmission prediction demonstrates that our two-step extract regression tree model adapted from the literature achieve similar accuracy as the black box neural network models while outperforming the commonly deployed, interpretable logistic regression models. Risk factors extracted via applying a regression tree to a neural network model were consistent with common readmission risk factors reported in the literature. This study suggests a possible way to improve the trust in machine learning based prediction models in clinical practice through the two-step prediction method, using readmission prediction as a case study. That is, by using regression trees extracted from the neural network model, instead of standard decision trees or logistic regression, we were able to significantly improve the accuracy without losing interpretability as compared to traditionally more powerful black box methods. This method may have broader applicability for accurate and medically interpretable predictions for other types of adverse events in health care.

## Electronic supplementary material

Below is the link to the electronic supplementary material.

Additional file 1: A supplement to the description of data and prediction performance of other machine learning models.

## Data Availability

The data that support the findings of this study are available from our collaborating hospital, but restrictions apply to the availability of these data, which were used under license for the current study, and so are not publicly available. Data are however available from the corresponding author upon reasonable request under non-disclosure agreement. Upon acceptance, we will publish all the code used in a GitHub repository.

## Abbreviations

ML: machine learning
AUC: Area Under the receiver operating characteristic Curve
AUPRC: Area Under the Precision-Recall Curve
HRRP: Hospital Readmissions Reduction Program
SLIM: Supersparse Linear Integer Model
BRL: Bayesian Rule Lists
IRB: Institutional Review Board
ROC: receiver operating characteristic curve
ACC: accuracy
MCC: Matthews correlation coefficient
SD: Standard deviation
GMM: Gaussian mixture model
LR: Logistic regression
SVM: Support vector machine
ET: Extremely randomized trees
LGBM: Light gradient boosting machine
XGB: Extreme gradient boosting
RF: Random Forest
NN: Neural network
DT: Decision tree
RT: Regression tree
