# Supplementary material for: Interpretable machine learning models for hospital readmission prediction: a two-step extracted regression tree approach
Source: BMC Med Inform Decis Mak. 2023 Jun 5;23:104. doi: 10.1186/s12911-023-02193-5 (PMC10243084; doi:10.1186/s12911-023-02193-5)
Supplement: Supplementary file 1 — Additional file 1: A supplement to the description of data and prediction performance of other machine learning models. [file 12911_2023_2193_MOESM1_ESM.docx]

Appendix

# Feature Distributions

As a supplement to the description of the data in the section **Materials and Methods: Study population**, Table S1 shows the descriptive statistics of the data. For the admission sources, we use abbreviations as: EM (Emergency Medicine), DO (Day Surgery), ES (Elective Surgery), DS (Day Surgery), EL (Elective Inpatient), SD (Social Overstay), DI (Day Surgery turn into Inpatient) and NB (Newborn). As the majority of patients were from the four specialties (medicine, surgery, orthopedics, cardiology), only patients from these four specialties were included in the analysis.

| Categorical Features | | | | | | | |
| --- | --- | --- | --- | --- | --- | --- | --- |
| Readmission (90 day) | | Discharge Location | | Admission Source | | Specialty | |
| Status 0 | 75.6% | Regular | 72.9% | EM | 54.0% | Med | 38.7% |
| Status 1 | 24.4% | Followup | 25.3% | DO | 7.7% | Ortho | 14.8% |
|  |  | Nursing Home | 1.8% | ES | 14.9% | Surg | 31.7% |
|  |  |  |  | DS | 10.0% | Cardio | 14.9% |
|  |  |  |  | EL | 5.7% |  |  |
| Gender | | Had operations | | SD | 6.1% |  |  |
| Male | 52.4% | Yes | 55.3% | DI | 1.5% |  |  |
| Female | 47.6% | No | 44.7% | NB | <.1% |  |  |
| Numeric Features | | | | | | | |
|  | LOS | Charlson score | Number of transfers | Age | van Walraven score | Prior visits |  |
| Min | 0 | 0 | 1 | 1 | -10 | 0 |  |
| Max | 206 | 0.7 | 78 | 111 | 1.72 | 12 |  |
| Average | 3.09 | 2.7 | 2.45 | 47.2 | 3.69 | 0.01 |  |
| Standard deviation | 6.78 | 14 | 2.14 | 22.9 | 35 | 0.15 |  |

Table S1. Feature distributions. Specialties limited to surgery, medicine, cardiology, and orthopedics.

# Additional Performance Scores for the Prediction Models

## Precision, Recall, F-value and MCC for Prediction Models

Table S2 shows precision (PR), recall, F-value and Matthews correlation coefficient (MCC) for the black box models and extracted trees. Extracted decisions trees and regression trees are denoted as E-DT and E-RT, respectively. Precision (positive predictive value) measures the proportion of positive predictions that were correctly identified by the model. Recall (or sensitivity, true positive rate) measures the proportion of actual positive cases that were correctly identified by the model. F-value is the harmonic mean of the sensitivity and the precision. MCC evaluates the performance of a binary classifier by taking into account both true positives, true negatives, false positives, and false negatives. It gives a score between -1 and 1 to indicate how good the classifier is, 1 being a perfect classifier and -1 being a totally wrong classifier.

| Model | | 30-day | | | | 90-day | | | |
| --- | --- | --- | --- | --- | --- | --- | --- | --- | --- |
|  |  | PR | Recall | F-value | MCC | PR | Recall | F-value | MCC |
| All Specialties | DT | 0.904 | 0.609 | 0.732 | 0.156 | 0.832 | 0.604 | 0.704 | 0.178 |
|  | LR | 0.908 | 0.726 | 0.813 | 0.241 | 0.849 | 0.731 | 0.797 | 0.294 |
|  | LGBM | 0.921 | 0.708 | 0.802 | 0.271 | 0.871 | 0.716 | 0.793 | 0.326 |
|  | ET | 0.924 | 0.657 | 0.778 | 0.231 | 0.873 | 0.662 | 0.754 | 0.290 |
|  | SVM | 0.917 | 0.705 | 0.798 | 0.239 | 0.851 | 0.670 | 0.746 | 0.266 |
|  | RF | 0.933 | 0.702 | 0.804 | 0.276 | 0.878 | 0.703 | 0.782 | 0.334 |
|  | XGB | 0.922 | 0.713 | 0.802 | 0.258 | 0.874 | 0.710 | 0.782 | 0.313 |
|  | NN | 0.905 | 0.732 | 0.813 | 0.233 | 0.865 | 0.663 | 0.751 | 0.279 |
|  | E-DT | 0.926 | 0.755 | 0.832 | 0.261 | 0.852 | 0.680 | 0.761 | 0.267 |
|  | E-RT | 0.925 | 0.752 | 0.830 | 0.263 | 0.856 | 0.709 | 0.774 | 0.271 |
| Main Specialties | DT | 0.897 | 0.569 | 0.698 | 0.101 | 0.801 | 0.578 | 0.672 | 0.114 |
|  | LR | 0.905 | 0.643 | 0.745 | 0.171 | 0.835 | 0.661 | 0.741 | 0.248 |
|  | LGBM | 0.918 | 0.671 | 0.775 | 0.221 | 0.848 | 0.673 | 0.749 | 0.273 |
|  | ET | 0.921 | 0.619 | 0.743 | 0.193 | 0.850 | 0.641 | 0.729 | 0.255 |
|  | SVM | 0.922 | 0.648 | 0.762 | 0.194 | 0.864 | 0.710 | 0.782 | 0.299 |
|  | RF | 0.916 | 0.643 | 0.752 | 0.211 | 0.856 | 0.645 | 0.738 | 0.272 |
|  | XGB | 0.920 | 0.621 | 0.739 | 0.182 | 0.837 | 0.646 | 0.726 | 0.239 |
|  | NN | 0.912 | 0.619 | 0.744 | 0.164 | 0.845 | 0.662 | 0.744 | 0.238 |
|  | E-DT | 0.917 | 0.605 | 0.730 | 0.195 | 0.847 | 0.632 | 0.735 | 0.254 |
|  | E-RT | 0.924 | 0.604 | 0.729 | 0.195 | 0.851 | 0.626 | 0.728 | 0.245 |

Table S2: Precision, Recall, F-value and MCC for the best black-box models and extracted trees.

## Confusion Tables for the Prediction Models

Table S3 shows the confusion tables for the best black-box models and extracted trees. A confusion table contains four numbers: from left to right, the first row reports the numbers of true positives and false negatives; the second row contains the numbers of false positives and true negatives.

|  | | 30-day | | | 90-day | | | |
| --- | --- | --- | --- | --- | --- | --- | --- | --- |
|  |  | ACC | AUC | AUPRC | ACC | | AUC | AUPRC |
| All Specialties | DT | 0.608 | 0.606 | 0.181 | 0.604 | | 0.603 | 0.288 |
|  | LR | 0.706* | 0.660 | 0.216 | 0.699* | | 0.664 | 0.339 |
|  | LGBM | 0.702 | 0.683 | 0.230* | 0.702* | | 0.685 | 0.355 |
|  | ET | 0.662 | 0.660 | 0.211 | 0.664 | | 0.668 | 0.335 |
|  | SVM | 0.696 | 0.661 | 0.215 | 0.661 | | 0.651 | 0.332 |
|  | RF | 0.698 | 0.688 | 0.232* | 0.697* | | 0.691 | 0.358* |
|  | XGB | 0.697 | 0.674 | 0.224* | 0.695 | | 0.678 | 0.348 |
|  | NN | 0.708* | 0.711* | 0.213 | 0.662 | | 0.713* | 0.329 |
| Main Specialties | DT | 0.574 | 0.573 | 0.158 | 0.571 | | 0.566 | 0.274 |
|  | LR | 0.636 | 0.621 | 0.180 | 0.652 | | 0.642 | 0.325 |
|  | LGBM | 0.669* | 0.655* | 0.200* | 0.664 | | 0.655 | 0.336* |
|  | ET | 0.625 | 0.638 | 0.188 | 0.643 | | 0.646 | 0.327 |
|  | SVM | 0.650 | 0.638 | 0.189 | 0.689* | | 0.670* | 0.341 |
|  | RF | 0.640 | 0.650 | 0.195 | 0.651 | | 0.657 | 0.334* |
|  | XGB | 0.626 | 0.631 | 0.184 | 0.641 | | 0.637 | 0.320 |
|  | NN | 0.619 | 0.654* | 0.178 | 0.649 | | 0.685* | 0.320 |
| Model | | 30-day | | | | 90-day | | |
| All Specialities | LGBM | \| [10351 \| 4215 \| \| --- \| --- \| \| 840 \| 1602] \| | | | | [9303 3674  1393 2638] | | |
|  | SVM | [10235 4331]  796 1646] | | | | [9125 3852  1290 2741] | | |
|  | NN | [10630 3936]  1030 1412] | | | | [8603 4374  1374 2657] | | |
|  | Extracted DT | [11003 3563  1017 1425] | | | | [8832 4145  1507 2524] | | |
|  | Extracted RT | [10949 3617  998 1444] | | | | [9210 3767  1630 2401] | | |
| Main Specialties | LGBM | [6250 3020  533 934]] | | | | [[5463 2645  952 1677] | | |
|  | SVM | [5904 3366  492 975] | | | | [5236 2872  872 1757] | | |
|  | NN | [5753 3517  565 902] | | | | [5370 2738  1028 1601] | | |
|  | Extracted DT | [5610 3660  474 993] | | | | [5128 2980  890 1739] | | |
|  | Extracted RT | [5602 3668  475 992] | | | | [5068 3040  900 1729] | | |

Table S3: Confusion tables for the best black-box models and extracted trees.

# Hyperparameter Settings

## Hyperparameter Settings for Random Forest

A random forest is composed of multiple decision trees, each of which is built from randomly selected features of the overall dataset. The results of these trees are averaged to give the final output. A preliminary random forest model was generated from the data set, and its performance indicated that it would be better equipped to make predictions for readmissions than logistic regression. For the all-specialty model, a baseline random forest model without any tuning performed with similar accuracy as the logistic regression, yielding an AUC of 0.60. Using the k-fold cross-validation method, we tuned the model hyperparameters, including the number of estimators, the way the data was split, the number of leaves in the trees, and the depths of the trees. These tuning procedures were optimized with a tuning package in Python to determine the ideal parameters that would also prevent overfitting. The tuned random forest model has the following hyper parameters: (1) the number of estimators is 100; (2) split measurement is Gini impurity; (3) the maximum depth of the tree is 6; (4) the minimum number of samples required to split an internal node is 15; (5) the minimum number of samples required to be at a leaf node is 5; and (6) the number of features to consider when looking for the best split is $\sqrt{n}$ where there are $n$ features in total.

## Hyperparameter Settings for Neural Network

A baseline neural network was created as a dense 9-layer fully connected Keras neural network. The model was run with a batch size of 10, and 5 epochs. This neural network had 5,169 trainable parameters. Without additional tuning, this model resulted in an AUC of 0.65 for predicting 90-day readmission. Using cross-validation, we further tuned hyper-parameters of the neural network, including number of hidden layers, batch size, number of neurons in each layer, and activation functions, among others. The tuned neural network predicting 30-day readmission for all specialties resulted in an AUC of 0.71. The 9-layer neural network performed the best with the original data. To improve the performance, we investigated the dataset and perform data processing including adding features of previous visiting times, grouping specialties, down sampling, etc. After these steps of data processing, the neural network with one hidden layer could achieve comparable performance with the 9-layer neural network. For the tree extraction, we used the 9-layer neural network because a more complex ML model is more likely to benefit from an extracted tree in our two-step approach for interpretability.

# Additional extracted decision trees and regression trees

Figures S1-S3 show additional decision trees and regression trees extracted from sampled data under different models, besides the regression tree shown in the section **Results: Machine learning modeling and performance**. The intensity of the blue (orange) color in a node corresponds to the proportion of examples classified as "readmitted" ("not readmitted").


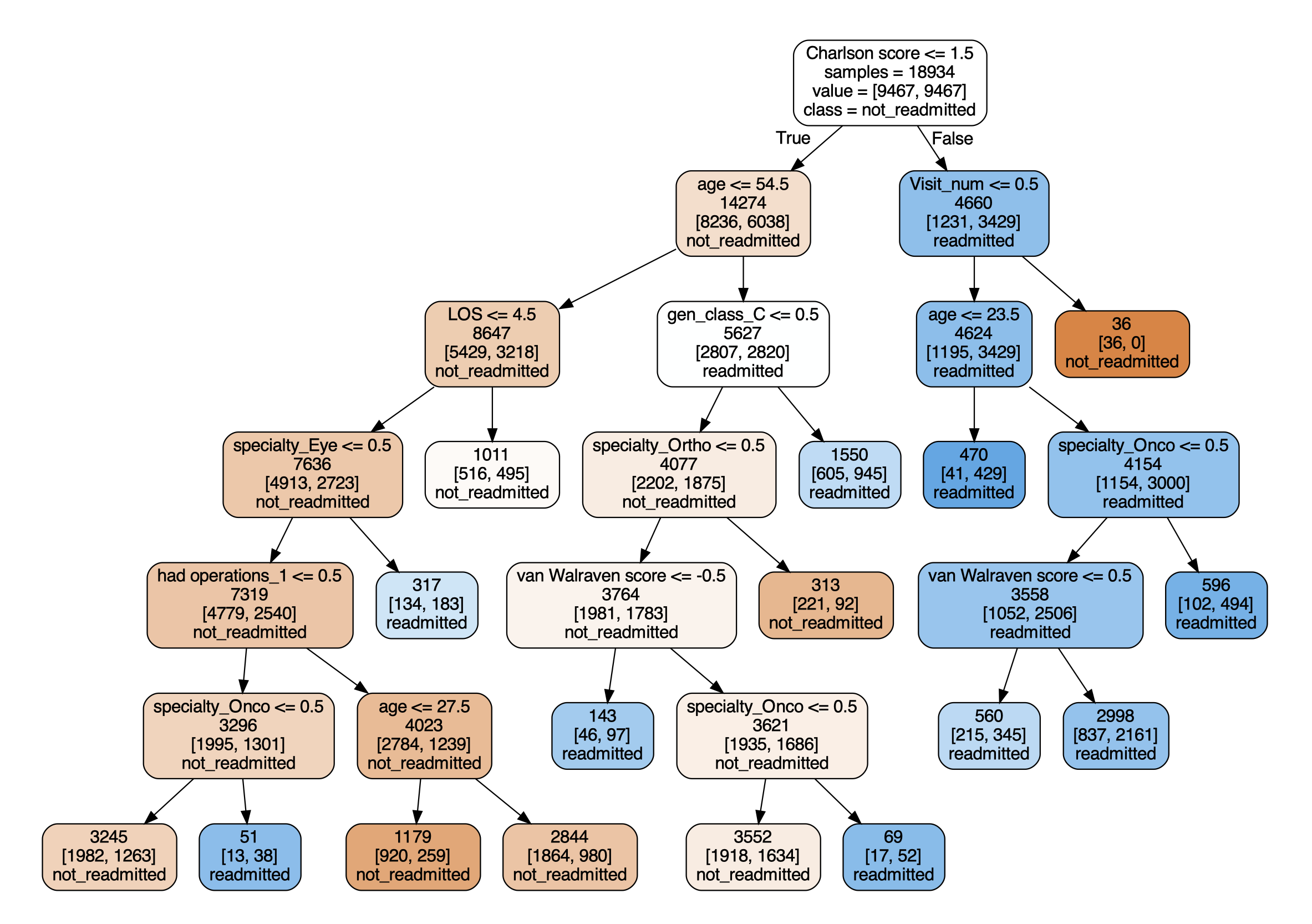


Figure S1: Decision tree extracted from sampled data (All specialties, 90-day readmission prediction).


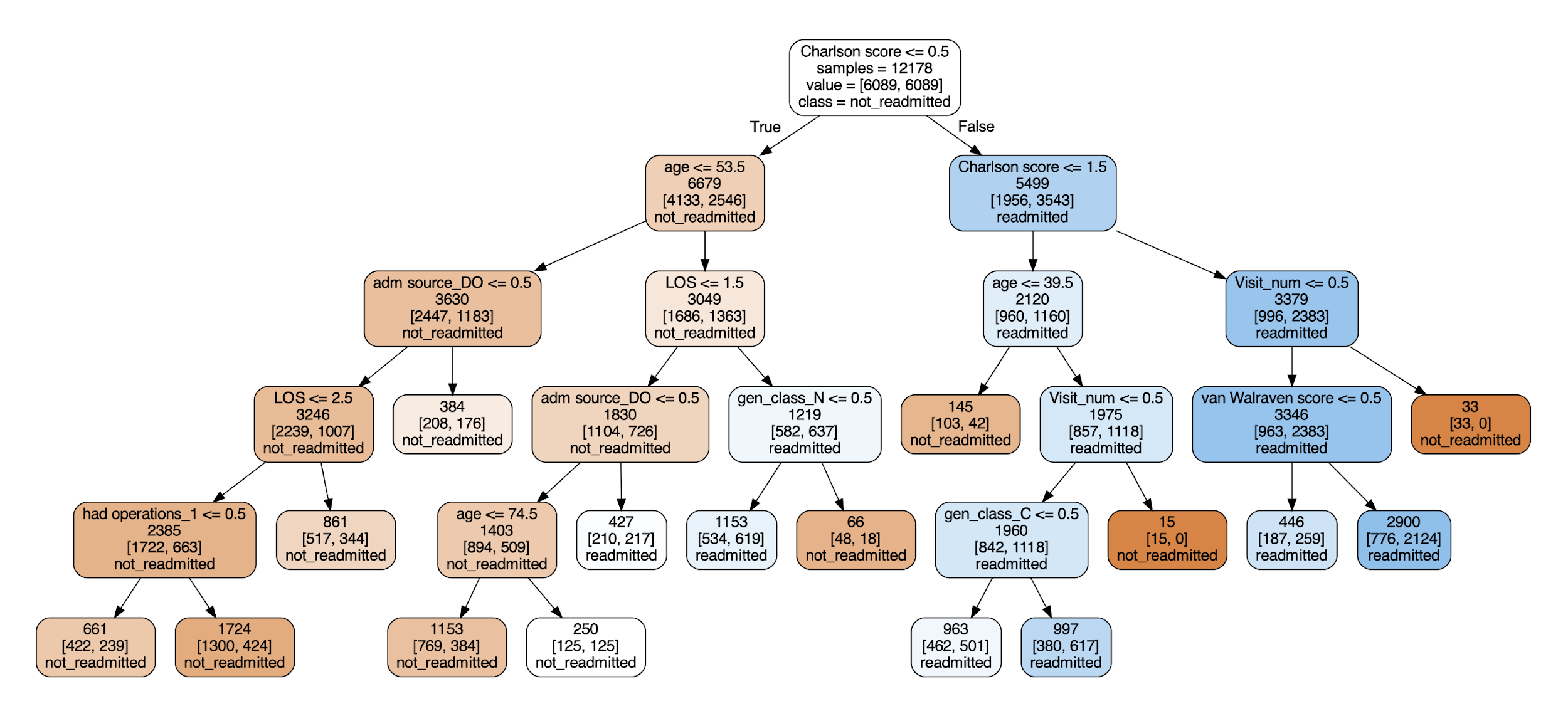


Figure S2: Decision tree extracted from sampled data (Four main specialties, 90-day readmission prediction).


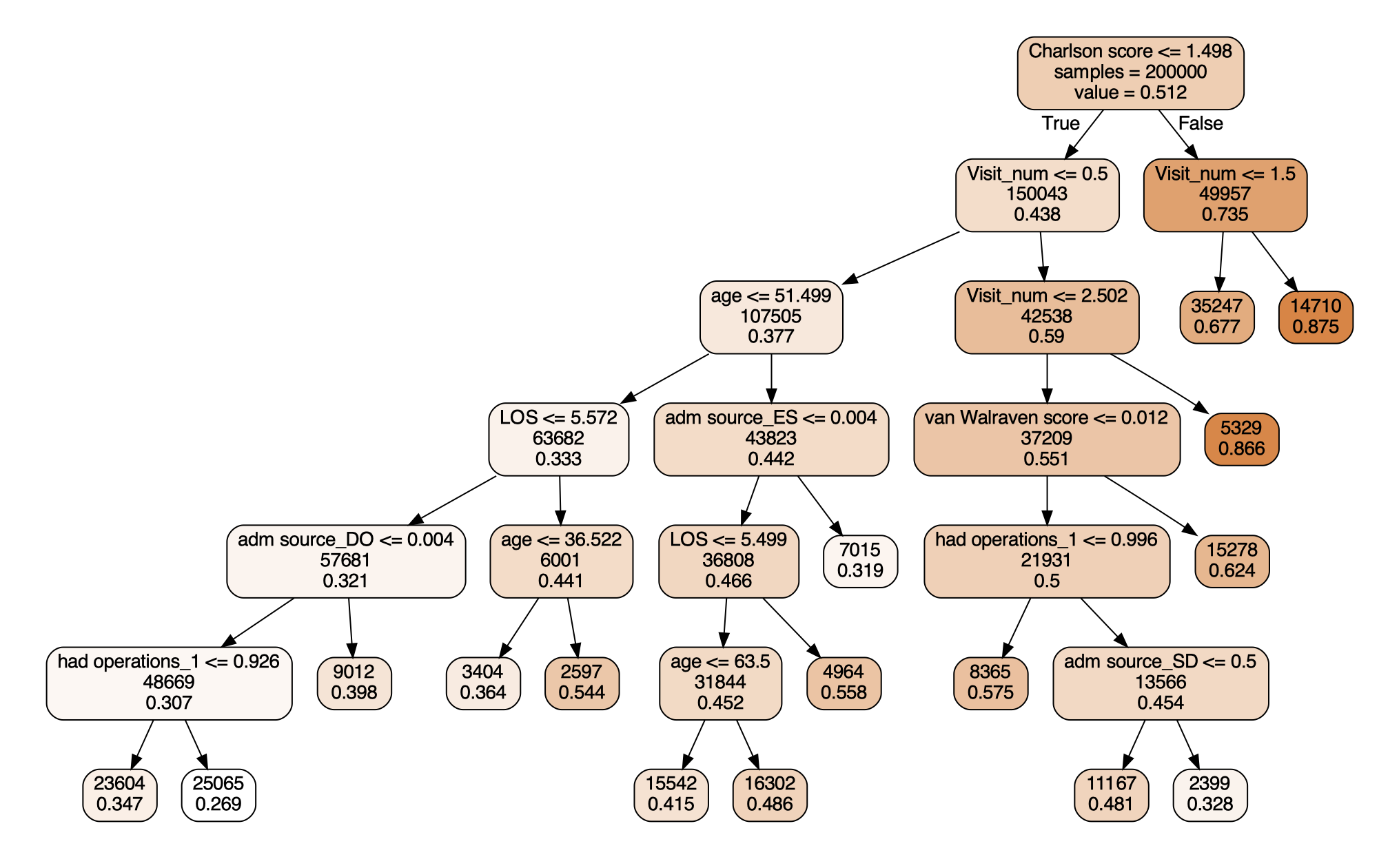


Figure S3: Regression tree extracted from sampled data (All specialties, 90-day readmission prediction).
